# Early neurocognitive outcomes following SEEG with radiofrequency thermocoagulation

**DOI:** 10.1002/epi4.70368

**Published:** 2026-10-19

**Authors:** Emily Cockle, Charles B. Malpas, Honor Coleman, Alissandra McIlroy, Joshua Laing, Martin Hunn, Matthew Gutman, Stacey Logan, Georgia‐Rose Robinson, Cecilia Harb, Catherine Meade, Wendyl D'Souza, Amy Halliday, Kristian Bulluss, Simon Vogrin, Rubina Alpitsis, Terence J. O'Brien, Genevieve Rayner, Andrew Neal

**Affiliations:** ^1^ Department of Neurology Alfred Hospital, Bayside Health Melbourne Victoria Australia; ^2^ Department of Neuroscience, School of Translational Medicine Monash University Melbourne Victoria Australia; ^3^ Melbourne School of Psychological Sciences University of Melbourne Parkville Victoria Australia; ^4^ Department of Medicine, Royal Melbourne Hospital University of Melbourne Parkville Victoria Australia; ^5^ Epilepsy Research Centre, Department of Medicine (Austin Health) The University of Melbourne Parkville Victoria Australia; ^6^ Bladin‐Berkovic Comprehensive Epilepsy Program, Department of Neurology Austin Health Heidelberg Victoria Australia; ^7^ Department of Neurosurgery Alfred Hospital, Bayside Health Melbourne Victoria Australia; ^8^ Department of Medicine St Vincent's Hospital Melbourne Victoria Australia; ^9^ Department of Neurosurgery St Vincent's Hospital Melbourne Victoria Australia; ^10^ Department of Surgery University of Melbourne Parkville Victoria Australia

**Keywords:** cognitive recovery, neuropsychology, RFTHC, stereo‐EEG

## Abstract

**Objective:**

Characterization of early neuropsychological outcomes following SEEG with radiofrequency thermocoagulation (RFTHC) is essential to guide patient expectations around return to work, study, and caregiving. The current study aimed to describe the early neurocognitive sequelae of SEEG.

**Methods:**

Consecutive patients undergoing SEEG were prospectively recruited from two tertiary epilepsy centers in Melbourne, Australia. Patients were administered the Repeatable Battery for the Assessment of Neuropsychological Status (RBANS) via telehealth before and 3 weeks after SEEG (*M* = 19.69 days, *SD* = 4.41). Given the telehealth platform, the coding subtest was substituted for the Symbol Digit Modalities Test, and a modified score of global cognitive function was calculated. To evaluate group changes, paired sample *t*‐tests were performed. Reliable change indices were computed to explore individual‐level changes. Pearson's correlation coefficients evaluated relationships between change in global cognitive function and SEEG admission variables. SEEG patients were invited to complete a semi‐structured interview canvassing the recovery following SEEG.

**Results:**

Twenty‐eight patients completed the neuropsychological examination (*M* = 37.89 years, *SD* = 9.72, range = 17–56 years, 54% female). All patients underwent RFTHC with a mean of 11.43 (SD = 6.87, range = 2–28) coagulation sites. At a whole group level, 3‐weeks post SEEG there was no evidence for global nor domain‐specific cognitive change. Reliable change indices with 90% confidence revealed only 4% (1/28) declined on at least two measures and 10% (3/28) showed psychometric improvement. Pearson's correlational coefficients showed no evidence for association between change in global cognitive function with SEEG factors including admission length, number of spontaneous or stimulated seizures, number of electrodes, or number of RFTHC sites. Subjective reports of language change tended to be accompanied by a decline on the RBANS language index that did not meet reliable change criteria. Most patients reported returning to work around 4 weeks post SEEG.

**Significance:**

By 3 weeks, gross cognitive functioning tends to be in keeping with pre‐SEEG performances for most patients. These findings have direct clinical implications for SEEG neuropsychological counseling.

**Plain Language Statement:**

SEEG is increasingly used to guide epilepsy surgery. Despite this, little is known about the cognitive recovery in the weeks following the procedure. We found that for most patients, broad cognitive functioning 3 weeks after SEEG with coagulation was consistent with function before SEEG, with only a small minority showing significant changes. Some people who reported language difficulties also showed a decline on language testing, although this was often small. Of those patients working before SEEG, most returned to work by 4 weeks. These results provide important evidence to guide patient counseling, recovery planning, and management after SEEG.

**Key points**

- Overall cognitive stability is seen in most patients 3 weeks following SEEG with RFTHC.
- Subjective reports of language decline following SEEG tended to be accompanied by nonsignificant decline on the RBANS language index.
- A majority of people returned to work around 4 weeks post SEEG with RFTHC.
- These results can directly inform pre‐SEEG neuropsychological counseling.

## 1 INTRODUCTION

Cognitive deficits are often a consequence of resective brain surgery.^1^, ^2^ Early recovery tends to be marked by a diffuse pattern of cognitive decline, commonly attributed to edema and compounded by fatigue and pain medications rather than reflective of permanent decline attributed to the resection site.^3^, ^4^, ^5^ During these initial weeks, prioritizing cognitive rest is recommended followed by a gradual return to cognitively demanding activities such as work and study.^6^ As these transient factors resolve, a more circumscribed neuropsychological profile emerges over the ensuing months, reflective of permanent deficits attributable to the underlying changes in brain networks.^7^, ^8^ Such neuropsychological deficits post epilepsy surgery tend to remain stable, persisting in some cases for follow‐up periods of 10+ years.^9^, ^10^, ^11^ Cognitve outcomes following stereoelectroencephalography (SEEG) have been less well described.^12^

SEEG is an invasive investigative procedure undertaken to assist with identification of the epileptogenic zone in patients with drug‐resistant epilepsy.^13^ Depth electrodes are implanted into the brain to record electrical activity, with the number and location of electrodes dependent on the hypothesized epileptogenic zone.^13^ To increase the likelihood of seizures during the admission, antiseizure medications are often reduced and sleep deprivation implemented.^13^ Cortical stimulation is also utilized to examine the epileptogenicity of implanted regions, and for mapping of neurological and neurocognitive functions.^14^ Toward the end of the admission, bipolar SEEG guided radiofrequency thermocoagulation (RFTHC) is often undertaken as standard of care in many centers. This involves delivery of a radiofrequency current between a pair of contacts on an electrode to create focal lesions within the suspected epileptogenic network.^15^, ^16^ While SEEG tends to be better tolerated than more invasive intracranial procedures,^17^ it can still be a mentally and physically taxing admission.

Emerging evidence suggests that for a majority of patients, SEEG and RFTHC are well tolerated from a cognitive perspective.^15^, ^18^, ^19^, ^20^, ^21^, ^22^, ^23^, ^24^ Some studies have suggested that larger non‐SEEG depth electrodes implanted into the hippocampus can be associated with memory decline, suggesting a micro‐lesional effect of the electrodes,^25^, ^26^ however, this has not been replicated in SEEG cohorts.^27^ By 3 months after SEEG with RFTHC, only a subset of patients appears to experience persistent neuropsychological deficits.^21^, ^27^, ^28^ At this stage, deficits tend to be highly specific to the cognitive functions supported by the coagulated brain network.^21^, ^27^ Specifically, we have previously shown that prospective cognitive follow‐up has found RFTHC within the dominant mesial temporal lobe to be associated with a decline in delayed verbal memory.^27^ Beyond documentation of transient neurological or speech and language impairments following SEEG‐guided RFTHC,^15^, ^17^, ^19^ the very early (<4 weeks) neuropsychological sequelae of SEEG has not been described. During this recovery stage, cognition may be impacted by SEEG‐related factors including the acute effects of edema, fatigue, and pain. This aligns with subjective recounts of the early recovery period post‐SEEG.^29^

Characterization of the early neuropsychological recovery post SEEG is important to inform preoperative counseling about the expected recovery trajectory and help guide planning around return to work, study, and caregiving. Additionally, understanding early recovery trajectories is essential to enable the recognition of unexpected cognitive decline and in turn identify patients in need of referral to timely intervention. Given significant differences between resective surgery and SEEG, recommendations surrounding early cognitive recovery cannot be directly translated between these procedures. As such, the current study aimed to prospectively delineate the early neurocognitive sequelae following a SEEG with RFTHC.

## 2 METHODS

### 2.1 Patients

For the neuropsychological examination, patients were recruited from The Alfred Hospital and St Vincent's Hospital Melbourne, Australia between September 2020 and December 2022. Inclusion criteria were: (1) over 16 years old (2) functional English to complete neuropsychological testing and (3) access to telehealth. Participants in the current study comprise a subset of those in previous studies published by our group.^21^, ^27^ All patients underwent a routine noninvasive pre‐SEEG workup, which included detailed clinical history, video‐EEG, FDG‐PET, fMRI, and neuropsychological evaluation. Participants who underwent SEEG at The Alfred were invited to participate in a subsequent study that involved a semi‐structured interview canvassing their experiences during the SEEG admission and recovery. This study was approved by The Alfred Hospital and St Vincent's Hospital's human research ethics committees, and all participants provided written informed consent.

### 2.2 Neuropsychological examination

Patients completed the Repeatable Battery for the Assessment of Neuropsychological Status (RBANS)^30^ via telehealth before SEEG and again 3 weeks post‐SEEG. This battery is designed for repeated administration over short periods of time, with alternative forms to reduce the impact of practice effects. The RBANS measures cognitive function with 12 subtests that can be administered in approximately 30 min. A total score and five index scores can be computed using age‐based normative data and have a mean of 100 and a standard deviation of 15. The index scores and associated subtests are: (1) Immediate Memory, which consists of the List Learning and Story Recall subtests; (2) Visuospatial Construction, which consists of the Figure Copy and Line Orientation subtests; (3) Language, which consists of the Picture Naming and Semantic Fluency subtests; (4) Attention, which consists of the Digit Span and Coding subtests; and (5) Delayed Memory, which consists of the List Recall and Recognition, Story Recall and Figure Recall subtests. The Symbol Digit Modalities Test (SDMT)^31^ was substituted for the Coding subtest to accommodate for telehealth administration as the patients did not have access to the Coding stimuli. As such, Digit Span was analyzed at a subtest level. This substitution prevented calculation of the RBANS total score. As such, a modified RBANS total score was uniquely developed for this study in collaboration with a statistician who is also a neuropsychologist (C.M.) by substituting the SDMT for the Coding subset (see Supplementary Material for Methods S1). This modified score is referred to as the score of global cognitive function. To capture any heterogeneity in cognitive outcomes, the RBANS is designed to assess 4/5 of the key cognitive domains (attention/processing speed, language, visuospatial function and memory) outlined by the International Classification of Cognitive Disorder in Epilepsy, the benchmark guidelines for neuropsychological assessment in epilepsy.^32^ It has been utilized in various neurological populations, including epilepsy, with good internal consistency, test–retest reliability, and convergent validity across index scores, with the exception of the convergent validity of the Attention index.^30^, ^33^, ^34^, ^35^, ^36^ Due to the multifaceted nature of the tasks that comprise the Attention index, subtest examination has been shown to be more sensitive for detecting change.^37^ The RBANS has been shown to produce comparable results across face‐to‐face and telehealth modalities.^38^, ^39^ The Test of Premorbid Function (TOPF) was administered as an estimate of premorbid function.^40^

### 2.3 Qualitative interview

Semi‐structured interviews exploring the SEEG admission and the recovery experience following SEEG were undertaken via teleconference. The interview was designed collaboratively by co‐authors guided by literature around psychosocial adjustment after epilepsy surgery.^41^, ^42^ Input was sought from a person with lived experience during a piloting stage. No major changes were made, and pilot data were included in the analysis. The final interview took approximately 60 min and comprised 15 open‐ended questions with prompts used to clarify or expand responses. The current study focuses on questions exploring cognitive recovery and return to work after SEEG.

### 2.4 SEEG admission

Electrodes were implanted under a general anesthetic on the day of admission. All patients were implanted with DIXI electrodes (8–18 contacts, 8 mm diameter and 2 mm contact length, Microdeep intracerebral electrodes; DIXI Medical, Becanson, France). All hippocampal electrodes were orthogonal, entering from the middle temporal gyrus that is not along the long axis of the hippocampus. Implantation with DIXI electrodes along this axis has not been associated with memory decline.^27^ Patients were then transferred to the neurosurgery ward. An individualized approach to antiseizure medication reduction and sleep deprivation was undertaken to increase the likelihood of spontaneous seizures. Cortical stimulation was typically undertaken around day 4 and included 2–3 sessions each lasting around 1–2 h. RFTHC and explantation were undertaken after sufficient seizures were captured. Patients underwent a CT‐brain the day following explantation and were discharged home following clearance from the neurosurgical team.

### 2.5 Radiofrequency thermocoagulation

The RFTHC technique is consistent with previously described methods^43^ and previously outlined in detail by our group.^27^ By way of summary, RFTHC was performed using a Cosman RF Generator via a Dixi selector box. Radiofrequency current was applied and voltage gradually increased to ~50 V for 30–60 s until a collapse of impedance was observed. In this study, each RFTHC was applied to a pair of contiguous contacts on a single electrode. The selection of contacts for RFTHC was determined through multidisciplinary consensus, based on the following criteria: (i) involvement in the epileptogenic zone or early seizure propagation; (ii) location within or at the boundary of an identified epileptogenic lesion; (iii) stimulation induced habitual electroclinical seizures at those contacts; (iv) a minimum distance of 2 mm from any blood vessel; and (v) not associated with primary eloquent cortex (i.e., primary motor) deficits on cortical stimulation. If a clinical response was elicited from the contact during cortical stimulation, benefits and risks were discussed.

### 2.6 Statistical analyses

#### 2.6.1 Neuropsychological assessment

Analyses were performed using JASP (Version 0.17.2.1)^44^ and IBM SPSS Statistics (Version 28.0).^45^ The Benjamini‐Hochberg procedure was used throughout to control for false discover rate (FDR) stemming from multiple comparisons, with *q* set at 5%. Where assumptions for parametric analyses were not met, nonparametric alternatives were used. Paired sample *t*‐tests were computed for the score of global cognitive function, the four RBANS index scores as well as the digit span subtest and SDMT. Pearson's correlation coefficients were computed to evaluate the relationship between change in the RBANS score of global cognitive function and SEEG admission variables. Reliable change indices (RCI) were calculated at 80% and 90% confidence intervals using Jacobson and Truax^46^ reliable change formula. Standard error of difference was calculated using the equation $S_{\text{diff}}=\sqrt{\left(\mathrm{SEM}1^{2}+\mathrm{SEM}2^{2}\right)}$, where *S*_diff_ is the standard error of difference, SEM1 the standard error of measurement at time 1 and SEM2 the standard error of measurement on retest. SEM1 was calculated using the equation $\mathrm{SEM}1=\mathrm{SD}\sqrt{1-r_{12}}$, where SD is the standard deviation from time 1 and *r*_12_ the test–retest reliability coefficient. A 90% RCI confidence interval was established by multiplying *S*_diff_ by 1.64. Confidence intervals were then adjusted to correct for practice effects (see Table S1).^47^ This analysis enables evaluation as to whether an individual's change across the review period on any one psychometric measure is significantly greater than measurement error alone.^46^

#### 2.6.2 Qualitative interview

Interviews were recorded, transcribed, and entered into NVivo 11 for reflexive thematic analysis.^48^ For the first two rounds of coding, authors SL and GR independently undertook inductive coding of transcripts to capture semantic and latent meaning, thereby minimizing expectancy effects in the early theme development. Responses pertaining specifically to the SEEG admission and recovery were selected for further analysis, with each phase analyzed separately as patients conceptualized them distinctly. Coding was completed iteratively, wherein transcripts were revisited to re‐code earlier sections considering newer codes. Initial themes were clustered and consolidated based on patterns of shared meaning to form higher‐order themes. Once a thematic map was established, the full dataset was then re‐examined against these themes for completeness and consistency. Reliability was established through sustained reflexivity throughout interviewing and analysis, with final themes and any coding discrepancies resolved via negotiated consensus within the team.^49^, ^50^ Functional recovery milestones were recorded verbatim in days/weeks post‐SEEG and tabulated with any qualitative details that aided interpretation.

## 3 RESULTS

### 3.1 Patients

Thirty‐three SEEG patients were considered for inclusion in the study. One was excluded due to insufficient English proficiency to complete the assessment without an interpreter, one due to lack of access to telehealth, and three declined participation (Figure S1). Demographic and clinical characteristics of the 28 patients who completed the neuropsychological examination are summarized in Table 1. The mean age of the sample was 37.89 (*SD* = 9.72) years. All patients underwent RFTHC toward the end of the SEEG admission. Between 2 and 28 coagulation sites were undertaken per patient with a mean of 11.43 (*SD* = 6.87). The mean length of stay in the hospital was 10.42 (*SD* = 3.57) days. Patients had a median of 6.50 (IQR = 2.75–9.25) spontaneous seizures and 1.00 (IQR = 0–3) stimulation‐induced habitual electroclinical seizures during the admission. All patients were taking the same antiseizure medications at the post‐RFTHC assessment compared to the pre‐RFTHC assessment. Mean time between pre‐and post SEEG RBANS assessment was 41.93 days (SD = 17.55, range = 24–78 days).

**TABLE 1:** Background demographic and clinical variables.

	All (*n* = 28)
Age, years, mean ± SD (range)	37.89 ± 9.72 (17–56)
Sex, F, *n* (%)	15 (54)
TOPF Index, mean ± SD (range)	95.61 ± 15.18 (68–131)
Age at epilepsy onset, years, mean ± SD (range)	15.48 ± 8.45 (1–37)
Duration of epilepsy, years, mean ± SD (range)	21.12 ± 12.74 (3–51)
Number of ASMs^a^, mean ± SD (range)	3.21 ± 0.99 (1–5)
Prior resection, *n* (%)	2 (7)
MRI lesion, *n* (%)	9 (32)
Type of lesion^a^	
Hippocampal sclerosis	3 (11)
Focal cortical dysplasia	3 (11)
Periventricular nodular heterotopia	2 (7)
Polymicrogyria	2 (4)
Other	3 (11)
Epileptogenic zone hemisphere, *n* (%)	
Dominant	11 (39)
Nondominant	13 (46)
Bilateral	4 (14)
Number of electrodes, *n*, mean ± SD (range)	13.67 ± 6.87 (7–19)
Length of admission, days, mean ± SD (range)	10.42 ± 3.57 (6–22)
Spontaneous seizures, *n*, mean ± SD (range)	18.25 ± 32.18 (0–101)
Stimulated habitual seizures, mean ± SD (range)	2.48 ± 3.04 (0–10)
RFTHC sites, *n*, mean ± SD (range)	11.43 ± 6.87 (2–28)
Time between RFTHC and assessment, days, mean ± SD (range)	19.69 ± 4.41 (12–31)
Seizure free at time of assessment	18 (64)

^a^ ASM at time of SEEG admission.

Of those who completed the early neuropsychological examination post SEEG, 22 underwent SEEG at The Alfred Hospital and were invited to complete the semi‐structured interview, with 13 participating in this subsequent study. The mean age of the sample was 37.89 (*SD* = 9.72, range = 24–51, female = 7, 54%) years. There was no significant difference between those who participated in the qualitative study and those that did not regarding age, sex, epilepsy duration, or surgical outcomes (*p* > .05, see Supplementary Material S1).

### 3.2 Early neuropsychological outcomes

Group‐level results across neuropsychological measures before and 3 weeks after SEEG are shown in Table 2. No statistically significant changes were observed on any RBANS index scores or the global cognitive function summary score.

**TABLE 2:** Neuropsychological examination before and 3 weeks after SEEG.

	Pre SEEG	Post SEEG	*p*	*p*_adj_	Cohen's *d*
Immediate memory	88.89 (14.96)	90.21 (18.71)	0.58	0.68	0.11
Visuospatial	97.21 (16.23)	94.54 (17.31)	0.41	0.59	0.16
Language	85.57 (17.35)	86.36 (12.85)	0.75	0.75	0.06
Delayed memory	89.75 (16.12)	93.82 (18.02)	0.10	0.59	0.32
Digit Span^a^	7.07 (2.94)	7.54 (3.16)	0.40	0.59	0.16
SDMT^b^	−1.30 (0.90)	−1.20 (1.12)	0.42	0.59	0.16
Score of Global Cognitive Function^c^	84.32 (11.84)	85.86 (14.01)	0.38	0.59	0.17

*Note*: RBANS total and index scores are reported as standard scores.

^a^ Scaled score.

^b^ z‐score.

^c^ Modified score.

Table 3 outlines individual‐level reliable change (see Supplementary Table 2 for baseline categorical neuropsychological data). Within the heterogeneous sample, no patterns between cognitive change and epilepsy‐related factors such as epilepsy syndrome, number of coagulations, or location for coagulation appear to emerge. Reliable change analysis with 90% confidence for RBANS index scores revealed that only 4% (1/28) of patients experienced a significant decline on two measures and 10% (3/28) an *improvement* on two measures. No patients experienced a decline or improvement on more than two measures. Reliable change analysis with 80% confidence for RBANS index scores revealed 14% (4/28) of patients experienced a decline on two measures, with no patients experiencing a decline on more than two measures. Of the four patients who declined on two measures, two had bitemporal epilepsy, one had dominant insula epilepsy, and one nondominant frontal epilepsy. There was no consistency in declined measures across these patients nor a clear association between declined cognitive domain and region coagulated. Fourteen percent (4/28) demonstrated an improvement on three measures. Reliable change for index scores is summarized in Table 4.

**TABLE 3:** RBANS individual reliable change.

	Age	Sex	RFTHC hemisphere	Epilepsy	MRI	Pathology	RFTHC no.	RFTHC sites	RBANS immediate memory		RBANS Visuospatial construction		RBANS language index		RBANS digit span		SDMT		RBANS delayed memory	
									80	90	80	90	80	90	80	90	80	90	80	90
1	48	Female	Dominant	Bilateral mesial temporal	Lesional	HS	16	Amygdala, HC, ERC, PHG	–	–	–	–	↓	–	–	–	–	–	↓	↓
2	45	Female	Nondominant	Insula	Non‐lesional		12	Insula, parietal operculum, Heschl's	–	–	–	–	–	–	↑	↑	–	–	–	–
3	41	Male	Dominant	Frontal	Non‐lesional		5	Pre SMA, prefrontal	↑	–	↑	↑	–	–	–	–	–	–	↑	↑
4	35	Female	Dominant	Anterior temporal	Lesional	PVNH, polymicrogyria	7	PVNH	–	–	↑	–	–	–	–	–	–	–	–	–
5	28	Female	Nondominant	Insulo‐opercular	Non‐lesional		19	SFG, MFG, pre SMA, premotor	–	–	–	–	–	–	–	–	–	–	–	–
6	24	Male	Nondominant	Occipital	Non‐lesional		5	Heschl's, planum temporale	↑	–	–	–	–	–	↓	–	–	–	–	–
7	42	Female	Nondominant	Mesial temporal	Lesional	HS	10	Amygdala, HC, ERC, TP	–	–	–	–	–	–	–	–	–	–	–	–
8	17	Male	Dominant	Tempro‐parieto‐occipital	Lesional	FCD, nodules	17	PVNH	↑	↑	↓	–	↑	↑	–	–	↑	–	–	–
9	27	Female	Dominant	Posterior frontal	Non‐lesional		2	SMA	–	–	↓	↓	–	–	↑	–	–	–	–	–
10	31	Male	Dominant	Temporal	Non‐lesional		13	Amygdala, HC, ERC, TP	–	–	↑	↑	–	–	–	–	–	–	–	–
11	47	Female	Dominant	Anterior lateral temporal	Non‐lesional		28	TP, MTG, ITG, STG, fusiform	–	–	–	–	↓	–	–	–	–	–	↑	↑
12	22	Male	Nondominant	Mesial temporal	Non‐lesional		18	Amygdala, HC, ERC, TP	↑	–	↓	–	↑	↑	↑	↑	–	–	–	–
13	42	Female	Nondominant	Anterior cingulate	Non‐lesional		3	Insula	↑	–	–	–	–	–	–	–	–	–	–	–
14	28	Male	Dominant	Bilateral Mesial temporal	Non‐lesional		4	Amygdala	–	–	↓	↓	–	–	–	–	↑	↑	–	–
15	43	Female	Nondominant	Mesial temporal and frontal	Lesional	HS	19	Amygdala, HC, TP, insula	↑	↑	–	–	–	–	–	–	–	–	–	–
16	43	Female	Nondominant	Posterior temporal	Non‐lesional		7	STG	–	–	–	–	–	–	–	–	–	–	↑	↑
17	41	Female	Dominant	Basal temporal	Non‐lesional		5	Fusiform, ITG	–	–	–	–	↓	–	–	–	–	–	–	–
18	38	Male	Dominant	Posterior insula	Non‐lesional		5	Insula	–	–	–	–	–	–	–	–	↑	↑	–	–
19	30	Male	Dominant	Mesial temporal	Lesional	HS	17	Amygdala, HC, ERC, PHG, fusiform	–	–	–	–	–	–	↓	↓	–	–	–	–
20	55	Male	Nondominant	Basal temporal	Non‐lesional		10	PHG, ITG, fusiform	–	–	–	–	–	–	↑	↑	–	–	–	–
21	33	Male	Dominant	Insula	Non‐lesional		8	Insula	–	–	↓	↓	–	–	↓	↓	–	–	–	–
22	51	Female	Nondominant	Hypothalamic Hamartoma	Lesional	Hypothalamic Hamartoma	2	Hypothalamic Hamartoma	–	–	↑	↑	–	–	–	–	↓	–	–	–
23	46	Female	Dominant	Mesial temporal	Lesional	PVNH	22	PVNH	↑	–	↓	↓	–	–	–	–	–	–	–	–
24	27	Male	Nondominant	Frontal	Non‐lesional		19	SFG	–	–	↓	–	↓	–	–	–	–	–	–	–
25	50	Female	Dominant	Bitemporal	Non‐lesional		9	ITG, MTG, fusiform	↓	–	–	–	–	–	–	–	↓	↓	–	–
26	33	Male	Dominant	Anterior frontal	Lesional	FCD	17	Orbitofrontal, middle rectus gyrus, rostral gyrus, amygdala	–	–	–	–	–	–	–	–	–	–	–	–
27	30	Female	Bilateral	Mesial temporal	Non‐lesional		14	Amygdala, HC, ERC, PHG	↓	↓	–	–	–	–	–	–	–	–	–	–
28	35	Male	Dominant	Prefrontal	Non‐lesional		7	Amygdala, HC, ERC, TP	–	–	–	–	–	–	–	–	–	–	–	–

*Note*: 80 indicated RCI change at 80% confidence, 90 indicated RCI change at 90% confidence.

Abbreviations: ERC, entorhinal cortex; HC, hippocampus; ITG, inferior temporal gyrus; MFG, middle frontal gyrus; MTG, middle temporal gyrus; PHG, parahippocampal gyrus; PVNH, periventricular nodular heterotopia; SMA, supplementary motor area; SFG, superior frontal gyrus; STG, superior temporal gyrus; TP, temporal pole.

**TABLE 4:** Reliable change indices.

	Improved RCI80	Declined RCI80	Improved RCI90	Declined RCI90
Immediate memory	7 (25)	2 (7)	2 (7)	1 (4)
Visuospatial	4 (14)	7 (25)	3 (11)	4 (14)
Language	2 (7)	4 (14)	2 (7)	0 (0)
Delayed memory	3 (11)	1 (4)	3 (11)	1 (4)
Digit span	4 (14)	3 (11)	3 (11)	2 (7)
SDMT	3 (11)	2 (7)	2 (7)	1 (4)

*Note*: *n* (%).

Pearson's correlational coefficients showed no association between change on the score of global cognitive function after SEEG with any SEEG admission factors including length of admission, *r*(28) = −.06, *p* = .75, *p*_adj_ = .92, number of spontaneous seizures, *r*(28) = −.26, *p* = .26, *p*_adj_ = .78, number of stimulated seizures, *r*(28) = .02, *p* = .92, *p*_adj_ = .92, number of electrodes, *r*(28) = .14, *p* = .47, *p*_adj_ = .78, or number of RFTHC sites, *r*(28) = −.14, *p* = .47, *p*_adj_ = .78.

### 3.3 Subjective cognitive change following SEEG

Six out of 13 (46%) patients who completed the semi‐structured interview endorsed changes in cognition following SEEG. Four out of 13 (31%) patients described a decline in language and/or speech function; exemplar descriptions are outlined in Table 5. For two people, this was an exacerbation of baseline difficulties, and for the remaining two, these were de novo difficulties. All four of these patients underwent RFTHC within the dominant hemisphere; one within the anterior lateral temporal region, one within the amygdala, one within the posterior insula, and one within the sensory motor area. All four patients reported ongoing speech or language difficulties to various degrees. Of note, none of these patients had undergone resective brain surgery the time of the qualitative interview. Of the four patients who reported subjective language changes following SEEG, one experienced a decline on the RBANS language index that met reliable change criteria with an 80% confidence interval, while two others experienced a decline on the RBANS language index that did not reach statistical criteria for reliable change.

**TABLE 5:** Patients reporting subjective speech and language decline post SEEG.

Case	RFTHC location	RBANS language profile	Categorization of decline	Example quotes
9	SMA	Declined 18 points on RBANS language index, not meeting RCI criteria	De novo speech difficulty	“I felt like I was slurring my words a little bit, and I still feel like I do that more than I used to”
11	TP, MTG, ITG, STG, fusiform	Declined 18 points on RBANS language index meeting RCI 80 criteria	Exacerbation of baseline language deficit	“There's a delay, in the word processing, and you'll know the actual term, where somebody says something to me, it has to be processed, interpreted, and then for me to be able to understand it and repeat or come up with an answer or a statement that's related to that. I think that is the most annoying part, actually… You feel a bit dumb.”
14	Amygdala	Declined 4 points on RBANS language index, not meeting RCI criteria	Exacerbation of baseline language deficit	“I have trouble finding the words to say sometimes”
18	Insula	No decline on RBANS language index	De novo language difficulty	“My speech was affected a like fair bit. I would go to say things, and I couldn't say it. I was basically retraining myself [to] take longer in my head before I could get it out.”

Abbreviations: ITG, inferior temporal gyrus; MTG, middle temporal gyrus; SMA, supplementary motor area; STG, superior temporal gyrus; TP, temporal.

Two additional patients described a change in processing speed immediately following SEEG that resolved over a period of weeks–months. One of these patients also described early severe fatigue following SEEG. No concurrent decline in psychometric measures of processing speed were apparent in these two people.

### 3.4 Return to work following SEEG

Nine out of 13 participants that completed the qualitative interview were employed at the time of SEEG. One reported returning to work 2 weeks post‐SEEG, four returned between 3–4 weeks post‐SEEG and two returned between 4–6 weeks post‐SEEG. Of the remaining two employed at the time of SEEG, one had an extended time away from work due to a career change and the other chose to wait until their hair grew back following SEEG before returning to work. Four of the 9 (44%) described a graded return to work.

## 4 DISCUSSION

The current study is the first prospective examination of early cognitive recovery following SEEG with RFTHC. By 3 weeks, at a whole‐group level the neuropsychological examination appeared consistent with pre‐SEEG assessment suggestive of broad cognitive stability. At an individual level, no patients demonstrated a psychometric decline on more than two neuropsychological measures. However, almost half (6/13) of the SEEG patients that completed the semi‐structured interview described cognitive changes, with language decline the most frequently reported. While subjective speech and language decline reportedly persisted, slowed processing speed post SEEG was described as transient. In a small subset of patients gainfully employed pre‐SEEG, early cognitive outcomes did not appear to unduly delay a return to their normal activities, with most returning to work around 4 weeks post SEEG with RFTHC.

Three weeks following SEEG with RFTHC there was no indication of diffuse cognitive deficits, with only two patients experiencing a decline on more than one measure when using 90% confidence reliable change criteria, and instead the majority demonstrating broad cognitive stability. Cognitive recovery at 3 weeks did not appear to be associated with any SEEG admission related variables including length of admission, number of seizures during the admission, number of electrodes, or number of RFTHC sites. Regardless of the demands of the SEEG admission, the pattern of cognitive stability at 3 weeks suggests this is sufficient time for broad cognitive recovery. This pattern of recovery occurs in the context of all patients undergoing RFTHC. As such, clinical implications likely extend to SEEG cases without RFTHC. Given that the SEEG admission is significantly less invasive than resective surgery, a more rapid broad cognitive recovery is not unsurprising.

There was a suggestion of subtle domain‐specific cognitive changes. Thirty‐one percent (4/13) of those interviewed described subjective language changes. While only one met statistical criteria for reliable decline in language, a trend of decline on the language index was observed in two further patients reporting subjective decline. Although the RBANS has been shown to detect changes in other neurological populations,^30^, ^33^, ^36^, ^51^ it has been found to be less sensitive to nuanced domain‐specific deficits compared with comprehensive neuropsychological batteries. Additionally, lesions created by RFTHC are significantly smaller than resective surgery,^15^, ^22^, ^52^ neuropsychological deficits may be less severe. As such, theoretically supported trends of decline on the RBANS should be carefully explored. All four patients reporting subjective change in language underwent RFTHC within the dominant hemisphere, suggestive of the contribution of an underlying organic etiology.^2^, ^53^, ^54^ Interestingly, two out of the four patients reporting subjective language decline demonstrated a statistical decline on a language measure 3 months post SEEG.^21^ This suggests that subtle decline on gross language screens such as the RBANS may be a clinical marker for ongoing deficits alongside subjective reports of change. Differences in SEEG patient recruitment between the current acute (3‐weeks post‐SEEG) sample and those that underwent a neuropsychological assessment 3 months post‐SEEG limited direct cross‐study comparisons.^27^

Findings from the current study have direct clinical implications for pre‐SEEG counseling and post SEEG monitoring. In helping patients to plan for their return to work or study, our results show broad cognitive stability at 3 weeks. In keeping with this finding, most SEEG patients interviewed were successfully able to return to work around 4 weeks post‐SEEG with RFTHC, oftentimes taking the form of a carefully graded return to usual duties and hours. While these preliminary findings provide useful information to guide return‐to‐work planning, they should be interpreted cautiously given the small sample size. Importantly, return to work planning should be individualized, balancing adequate time to recover in the weeks following discharge from an admission that can be physically and emotionally challenging with medication reductions, sleep deprivation, spontaneous and stimulated seizures as well as complicated decisions being made about epilepsy management.^13^ As such, it is essential to consider any ongoing fatigue or pain symptoms when planning a return to work as well as the patients workplace including job requirements, support available, and capacity for a graded return. As outlined by a lived experience interviewee, consideration of changes in physical appearance (i.e., hair being shaved) is also important to consider when planning a return to work.

Furthermore, this study has important implications for neuropsychological rehabilitation. Understanding that broad cognitive stability is expected by 3 weeks provides valuable clinical context to aid interpretation of any deficits. Changes on a gross screen of cognition such as the RBANS should thus be a marker to prompt early intervention. The limited sensitivity of the RBANS to detect subtle yet functionally meaningful changes, however, should be considered.^55^, ^56^ Subjective reports of cognitive change should always be investigated with a comprehensive neuropsychological evaluation. Finally, the findings highlight the role for cognitive rehabilitation in individuals who report early language changes, as these difficulties may persist over time.^12^ Together, this emphasizes the importance of neuropsychological review post SEEG to improve patient outcomes.

This study is limited by its modest sample size, which precluded subgroup analyses according to the anatomical region of RFTHC. Given the heterogeneity in RFTHC location, domain‐specific cognitive decline was likely obscured in whole‐group analyses. Prior studies have also described verbal memory decline following depth electrode implantation into the hippocampus, with histopathology of a case study revealing associated structural damage within the parahippocampal cortex.^25^, ^26^ Subgroup analysis remains a critical area for future investigation, particularly given indications of verbal memory decline following RFTHC within dominant mesial temporal structures^27^ and the potential for SEEG electrodes to cause micro lesions.^57^ To address this limitation, individual reliable change analyses were also examined in the current study. Given the use of a cognitive screening measure, it is unsurprising that patterns between domain‐specific neurocognitive deficits and regions coagulated were not identified. While this has been the focus of our previous work with comprehensive neuropsychological assessments,^21^, ^27^ the current study instead aimed to capture broader patterns of cognitive recovery during the early post‐SEEG period. Furthermore, the impact of mood on subjective and objective cognitive outcomes was not considered. This remains an important area for future research.

Additionally, the RBANS required adaptation for telehealth administration, which may introduce variability. As outlined, while sufficient to capture gross cognitive change, this commonly used cognitive screen may lack sensitivity to detect subtle cognitive change. Future research should prioritize the development of more sensitive cognitive screening measures for people with epilepsy. Executive function should also be considered in future cognitive screens, as it is the only cognitive domain not captured within the RBANS. Lastly, validation for telehealth delivery is essential to ensure equitable access for patients unable to attend in‐person assessments.

In summary, this study is reassuring that for most people SEEG with RFTHC is accompanied by early cognitive stability. By 3 weeks, broad cognitive functioning appears to be in keeping with pre‐SEEG baseline performances, although the possibility of subtle difficulties cannot be entirely excluded.

## FUNDING INFORMATION

AN was supported by National Health and Medical Research Council (NHMRC) Grant (APP2009152). GR was supported by NHMRC Grant (APP2008737) and Australian Epilepsy Research Fund. TJO was supported by NHMRC Grant (APP1176426).

## CONFLICT OF INTEREST STATEMENT

None of the authors has any conflict of interest to disclose. We confirm that we have read the Journal's position on issues involved in ethical publication and affirm that this report is consistent with those guidelines.

## ETHICS STATEMENT

The Alfred Hospital and St Vincent's Hospital's human research ethics committees and all participants provided written informed consent.

## Supporting information


Figure S1:

## Data Availability

The data that support the findings of this study are available on request from the corresponding author. The data are not publicly available due to privacy or ethical restrictions.
