# Supplementary material for: Early neurocognitive outcomes following SEEG with radiofrequency thermocoagulation
Source: Epilepsia Open. 2026 Oct 19:10.1002/epi4.70368. Online ahead of print. doi: 10.1002/epi4.70368 (PMC13680816; doi:10.1002/epi4.70368)
Supplement: Supplementary file 1 [file EPI4-9999-0-s001.docx]

**Supplementary Material**

*Prorating the RBANS Global Score*

SDMT oral raw scores were converted to z-scores using age and education matched normative data (reference for norms). The z-scores were then converted to standard scores in line with the coding index on the RBANS. The standard score could then be used to obtain an associated RBANS Coding raw score. If the manual provided a range of raw scores for a given scaled score, the mean was used. The Coding raw score could then be used with the Digit Span raw score to calculate the Attention Index score. The prorated Attentional Index Score could then be summed to the remaining 4 Index Scores to obtain a Total Score. As outlined in the RBANS manual, the Total Score was then converted to a Standard Score. This was used as a prorated Total Score in our study.

**Recruitment bias analysis**

There was no significant difference in participation in the qualitative interview following completion of the RBANS study and age, *t*(20)=0.55, *p*= .59, sex, χ² (1,22) = 0.32, *p* = .57, epilepsy duration, *t*(20)=0.18, *p*= .86, or surgical outcome post SEEG χ² (1,22) = 0.03, *p* = .86.

Supplementary table 1: reliable change analysis

|  | Test retest reliability | SD1 | SEM1 | SD2 | SEM2 | Sdiff | RCI90 |
| --- | --- | --- | --- | --- | --- | --- | --- |
| Immediate memory | .68 | 12.9 | 7.30 | 13.2 | 7.47 | 10.44 | 17.12 |
| Visuospatial | .65 | 13.2 | 7.81 | 12.9 | 7.63 | 10.92 | 17.91 |
| Language | .46 | 14.2 | 10.43 | 14.9 | 10.95 | 15.12 | 24.80 |
| Delayed memory | .64 | 14.0 | 8.40 | 15.8 | 9.48 | 12.67 | 20.77 |
| Digit Span | .73 | 3.5 | 1.82 | 3.7 | 1.92 | 2.65 | 4.35 |
| SDMT | .80 | 8.69 | 3.89 | 9.47 | 4.24 | 5.75 | 9.43 |

Supplementary figure 1: Participant recruitment and inclusion flow diagram

Supplemental Table 2. Pre SEEG Neuropsychological Assessment Categorical Score

|  | RBANS Immediate memory | RBANS  Visuospatial Construction | RBANS Language Index | RBANS Digit Span | SDMT | RBANS Delayed Memory |
| --- | --- | --- | --- | --- | --- | --- |
| 1 | Extremely Low | Low Average | Extremely Low | Average | Borderline | Borderline |
| 2 | Very High | Very High | Average | Extremely Low | Average | Very High |
| 3 | Low Average | Borderline | Average | Borderline | Low Average | Extremely Low |
| 4 | Average | Low Average | Low Average | Extremely Low | Extremely Low | Average |
| 5 | Borderline | High Average | Extremely Low | Low Average | Low Average | Average |
| 6 | Average | High Average | Low Average | Low Average | Borderline | Average |
| 7 | Average | Average | Average | Borderline | Borderline | Low Average |
| 8 | Average | Average | Extremely Low | Low Average | Borderline | Borderline |
| 9 | High Average | Average | Average | Extremely Low | Borderline | Low Average |
| 10 | Low Average | Average | High Average | Average | Average | Low Average |
| 11 | Low Average | Average | Average | Low Average | Borderline | Borderline |
| 12 | Low Average | High Average | Extremely Low | Average | Low Average | Average |
| 13 | Average | Very High | Average | Average | Average | Average |
| 14 | Low Average | Average | Borderline | Borderline | Extremely Low | Average |
| 15 | Borderline | Average | Average | Average | Average | Average |
| 16 | Borderline | Extremely Low | Average | Low Average | Extremely Low | Borderline |
| 17 | High Average | Low Average | Average | Average | Average | High Average |
| 18 | Low Average | Average | Borderline | Average | Average | Low Average |
| 19 | Average | Average | Extremely Low | Very High | Borderline | Average |
| 20 | Average | Average | Low Average | Low Average | Average | Average |
| 21 | Average | Average | Low Average | Average | Average | Average |
| 22 | Average | Low Average | Average | High Average | Average | Average |
| 23 | Extremely Low | Average | Borderline | Low Average | Extremely Low | Extremely Low |
| 24 | Borderline | Low Average | Average | Average | Low Average | Low Average |
| 25 | Low Average | Borderline | Extremely Low | Borderline | Borderline | Average |
| 26 | Borderline | Extremely Low | Low Average | Average | Borderline | Average |
| 27 | Borderline | Average | Average | Average | Low Average | Extremely Low |
| 28 | Average | Very High | Borderline | Low Average | Low Average | Average |
